# Supplementary material for: Astrocytes Modulate Somatostatin Interneuron Signaling in the Visual Cortex
Source: Cells. 2022 Apr 20;11(9):1400. doi: 10.3390/cells11091400 (PMC9102536; doi:10.3390/cells11091400)
Supplement: Supplementary file 1 [file cells-11-01400-s001.zip › cells-1686761-supplementary.pdf]

cells

MDPI

Supplementary Material

Astrocytes Modulate Somatostatin Interneuron Signaling in the Visual Cortex

Vanessa Jorge Henriques [1,2], Angela Chiavegato [2], Giorgio Carmignoto [1,2,†] and Marta Gómez-Gonzalo [1,2,*,†]

[1] Neuroscience Institute, National Research Council (CNR-IN), 35131 Padua, Italy; vanessajhenriques@gmail.com (V.J.H.); gcarmi@bio.unipd.it (G.C.)
[2] Department of Biomedical Science, University of Padua, 35131 Padua, Italy; angela.chiavegato@unipd.it
* Correspondence: marta.gomezgonzalo@cnr.it; Tel.: +39-049827-6041/6075
† These authors contributed equally to this work.

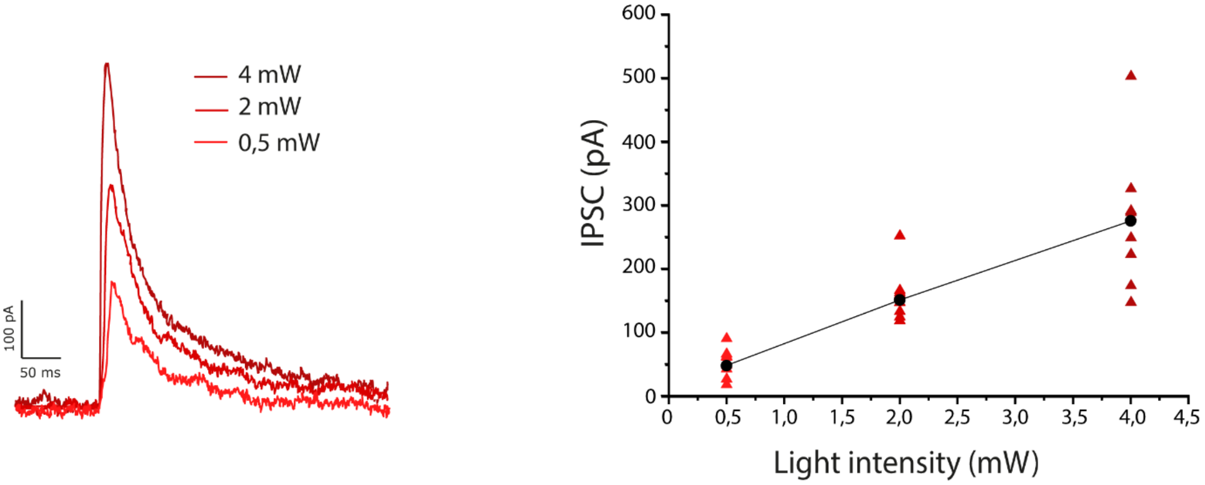

Figure S1: IPSC amplitude recorded from layer II-III PNs (*n* = 10) as a function of light pulse intensity.
